# Mechanisms of impairment in hair and scalp induced by hair dyeing and perming and potential interventions

**DOI:** 10.3389/fmed.2023.1139607

**Published:** 2023-05-18

**Authors:** Yongyu He, Yu Cao, Binji Nie, Junpu Wang

**Affiliations:** ^1^Department of Pathology, Xiangya Hospital, Central South University, Changsha, China; ^2^Xiangya School of Medicine, Central South University, Changsha, China; ^3^Department of Pathology, School of Basic Medicine, Central South University, Changsha, China; ^4^National Clinical Research Center for Geriatric Disorders, Xiangya Hospital, Central South University, Changsha, China

**Keywords:** hair dyes, hair perms, mechanisms of impairment, allergic contact dermatitis (ACD), interventions

## Abstract

With the rapid growth of beauty and personal care industries, many hair-relevant products, hair dyes and hair perms in particular, are increasingly prevalent in both women and men, regardless of being young or old as they frequently change hair color or shape to enhance youthfulness and beauty and to follow fashion trends. Hair dyes and perms alter hair color and/or shape by mechanically changing the physical structure and chemical substances of the hair shaft. However, treatment of hair with chemical formulations has been potentially ascribed to adverse outcomes in the hair shaft including structure damage, chemical constituent disorder, and impaired physical properties, although hair cosmetics procedures are intrinsically safe. Nevertheless, the mechanisms of impairment in the hair shaft and scalp induced by hair dyeing and perming remain elusive. Additionally, adverse reactions activated by exposure to specific chemical ingredients including skin irritation, allergic contact dermatitis (ACD), and even cancer risk have been reported clinically, but existing evidence is not consistent enough in the case of human studies. Herein, the review aims to give an overview of hair cosmetics, especially concerning the basic knowledge about various hair dyes and perms, the consequences for hair shafts and the scalp resulting from the application of hair cosmetics mentioned above, mechanisms of hazardous outcomes, and potential desirable interventions to alleviate the impairment.

## Introduction

In recent history, people have become increasingly focussed on the self-image that they project to others, which makes a difference in how they are perceived, particularly now, when pursuing beauty is getting more prevalent (1). People use a variety of cosmetics mainly to beautify their appearances, among which hair cosmetics are representatives (2). Hair, for example, is an indicator of attractiveness and beauty and also a fantastic medium to make a statement of one's femininity or masculinity (1, 3). Irrespective of gender, age, economic income, and social status, people embellish their hair through chemical techniques, emphasizing the importance given to appearance. People in modern societies can easily handle and alter their hairstyle from time to time through bleaching, dyeing, and perming (4), which require various natural and/or artificial chemical components of hair cosmetics and promote an increasing search for chemical hair transformations globally. Statistics data estimated that in the United States and Europe, 50%−80% of women and 10% of men aged 40 years and above have the experience of dyeing their hair and have analyzed the tendency of hair dye use which remained stable over the past decades (4). The color and shape of one's hair can speak volumes. The current global hair dyes and perms industry's market value is profitable, among which the market value of hair dye/color worldwide is ~30 billion US dollars and is predicted to be ~42 billion US dollars by 2025 (1). Further research concerning the rapidly growing hair cosmetics industry is ongoing.

Hair colorants come in two forms: oxidative (permanent) and non-oxidative (semi-permanent and temporary), in which the ingredients vary a lot. Hence, the manipulation mechanisms of different kinds of hair dyes are diverse. Permanent hair dyes bleach and add a new color to hair through the penetration of smaller dye precursors into the cortex and subsequent oxidation (3), while permanent hair wavings convert straight hair into curly through a procedure comprising some steps that involve breaking and restructuring the hair disulfide bond (4). Nevertheless, hair cosmetics such as hair dyes do damage to the hair shaft and scalp, which is revealed by the data on hair dye safety monitored by the World Health Organization's International Agency for Research on Cancer and the US Food and Drug Administration (5). Likewise, some of the chemicals in hair dyes and hair perms are probably carcinogenic to those who are exposed to them occupationally such as hairdressers and barbers. Significant findings have been made in this field, and newer studies about the application, outcomes, and interventions for adverse effects in the hair shaft and scalp keep improving.

However, personal use of hair cosmetics poses the greatest potential concern on public health implications including impairment in hair shaft: structural damage, chemical constituent disorder, and impaired physical properties, as well as skin irritation and allergic contact dermatitis (ACD) and even carcinogenic hazard (5). Scientists have tried to determine the association between hair cosmetics and health risks considering the prevalence of hair care products around the world. Consequently, monitoring the hazardous effects of hair cosmetics by developing more non-toxic, mild, and biocompatible pathways for beautifying hairstyles is ongoing. This review aims to provide contradictory or controversial knowledge about the use of hair cosmetics, which involves exploring mechanisms of impairment in hair and scalp induced by hair dyeing and perming and interventions for such impairment.

## Damage to the hair and scalp caused by dyeing or perming hair

Personal use of hair dyes and hair perms threatens the health of the hair shaft, scalp, and even modifications of genetic variation in different types of cells. The whole hair-dyeing and hair-shaping process can be roughly divided into two procedures according to the two main chemical ingredients involved in hair dyes and perming agents: the physical breakage of the primary constituent of hair and the addition of precursors/couplers (6). The former step is known to initiate the hair-dyeing and hair-shaping process by using the developer, in which hydrogen peroxide oxidants are usually involved. PPD and related para-amino compounds, such as p-aminophenol and cocamidopropyl betaine, are regarded as the real allergens inducing allergic contact dermatitis, and as an occupational respiratory disease (7, 8). Certain substances in precursors/couplers such as aromatic amines have a skin-sensitizing nature and have been suggested as possible carcinogens or mutagens in experimental studies, and for example, chemicals p-phenylenediamine and aminophenyl are associated with human cancer risk. Published epidemiological studies and experimental studies may summarize the findings (9). The degree and permanence of the damage induced by different products such as hair dyes and perms are analyzed as follows.

### Knowledge of hair dyes and perming agents

Hair dyes are most commonly classified into temporary dyes, semi-permanent dyes, and permanent dyes (3), depending mechanically on the composition of a dye (oxidative or non-oxidative) as well as its depth of penetration of the hair shaft and literally according to the coloring time they maintain (10). In addition, coloring agents can be divided into two general forms including oxidative (permanent) and non-oxidative (semi-permanent and temporary) owing to dyeing principles. Hair dyes embellish the hair by bleaching and/or adding a new color to it briefly and permanent hair dyes are among the most frequently used cosmetic products on the market. The vital ingredients of permanent hair dyes consist of primary intermediates, such as phenylenediamines [e.g., p-phenylenediamine (PPD) and toluene-2,5-diamine (PTD)] and p-aminophenols, and couplers (e.g., m-aminophenols and m-hydroxy phenols), as well as the presence of peroxide (3, 11). During the hair-coloring process, phenylenediamines recognized as precursors and couplers together penetrate the loose hair, and subsequently, redox reactions occur under the activation of oxidants. The combination of oxidizing and alkaline agents in permanent hair dyeing induces swelling of the hair cuticle, which facilitates the diffusion of the colorless phenylenediamines into the hair cortex. The colorless precursor removes the natural color of the hair and its following oxidation turns itself into large colored molecules, which adds new color to the hair. On the other hand, semi-permanent hair colorants contain an alkaline agent (e.g., ethanolamine and sodium carbonate) and a reduced level of hydrogen peroxide relative to permanent hair dyes. Temporary hair dyes include coloring compounds that stain hair directly by enclosing large molecular pigments into the hair fibers. Moreover, with the sustained growth of the hair cosmetics industry globally, various natural botanic and artificial chemical compounds are involved in hair colorants (12).

P-phenylenediamine (PPD) and toluene-2,5-diamine (PTD) play crucial roles in hair dyeing, but mounting evidence has demonstrated them to be toxic and hazardous components of hair dyes (3). Most formulations of hair dyes contain PPD, a well-known skin sensitizer (13). PPD is a preferred constituent in many other coloring products such as textile (or fur) dyes, photographic developers, oils, and gasoline (3). Extensive case reports suggest that PPD is the cause of allergic contact dermatitis (ACD) as well as other complications, and the easing frequency of positive patch test reactions to PPD further identifies it as an ACD allergen (14). Moreover, animal models such as the local lymph node assay (LLNA) and the guinea pig maximization test (GPMT) have been widely used to predict the identification of PPD sensitization (13). Epidemiological studies on consumers' allergies to p-phenylenediamine (PPD) conducted by Thyssen et al. estimated the median prevalence of PPD sensitization among dermatitis patients was 4.3% in Asia, 4% in Europe, and 6.2% in North America, and allergic tendencies had a widespread increase among Asian dermatitis patients, slight fluctuation in Europe, and continued high prevalence in North America (15). Skin irritation and even ACD lead to the major health burden of PPD-containing hair care products and financial repercussions. PTD is one of the most frequently used formulations in oxidative hair dyes though little is known about immune responses to PTD-containing permanent hair colorants. The US Food and Drug Administration (FDA) showed that toluene-2,5-diamine and the sulfate salt (toluene-2,5-diamine sulfate) are used in a total of 79 and 168 products, respectively (16). Immune responses to hair dyes containing toluene-2,5-diamine (PTD) are concentration-dependent, and they are reflected in the local inflammatory response occurring in the skin and draining lymph nodes (11). In detail, permanent hair dye products containing 1.60% of PTD induced strong local inflammation and caused T- and B-cell infiltration and proliferation as well as an increased number of regulatory T cells in the draining lymph nodes while permanent hair colorants containing 0.48% of PTD induced skin inflammation but only minor responses in the draining lymph nodes.

Similarly, a growing number of people selectively perm their hair, emphasizing the importance given to appearance. Perming agents can be divided into cold ironing agents and hot ironing agents. The manipulations involve oxidizing and reducing steps that necessitate the use of multiple reactive components of perming agents including reductant, alkali agent, surface active agent, stabilizer, oxidant, and oily components (8). The reductants cut the disulfide bond between hair proteins, making hair soft and easy to sculpt. An alkali agent is used to increase the pH value of the perming products, improve the reduction ability to reduce the agent, and at the same time separate the hair cuticle so that other ingredients can reach the hair cortex. The surfactant can play roles in infiltration and emulsification, increasing the moisture of the hair. Stabilizers prevent the oxidation of reducing agents by metal ions. After the initial plasticity is completed, the oxidant repairs the disulfide bonds that have been cut in the hair, holding the curly hair in place. Oily ingredients can be added to the head for their repair effect, making the hair shiny and moisturized. Current research in the literature reported detrimental outcomes such as hair breakage, skin irritation and sensitization concerning hazardous ingredients, and misuse of permanent wavings (4). A common surfactant in perm wave solutions is cocamidopropyl betaine (CAPB), and patch test records at the Finnish Institute of Occupational Health revealed that CAPB causes contact allergy, while a number of studies have indicated that the impurities in CAPB such as dimethylaminopropylamine (DMAPA) and cocamidopropyl dimethylamine (cocamidopropyl-DMA) induce dermatitis (17) (Table 1).

**Table 1:** A list of the major ingredients in prevalent hair products (hair dyes and hair perms).

	**Compositions**	**Health effects**	**Function**	**References**
Hair dyes	p-Phenylenediamine (PPD)	Mild contact dermatitis: scalp dermatitis, periorbital dermatitis, face, and neck dermatitis, chronic actinic dermatitis severe life-threatening events: angioedema, bronchospasm, asthma, renal impairment	Primary intermediate	(18, 19)
	Toluene-2,5-diamine (PTD)	Ear swelling, cell infiltration, T- and B-cell infiltration, and proliferation in the draining lymph nodes (CD4^+^ CD25^+^ FoxP3^+^ Treg cells within the draining lymph nodes correlated with this)		(11)
	p-aminophenol	Facial/scalp rash, edema of the eyelids, forehead, or neck, eczema of the scalp or neighboring skin, facial swelling, or even a more generalized rash		(20)
	aminophenol/m-hydroxyphenol	Skin sensitization	Oxidative coupler	(3)
	Resorcinol	Moderate facial/scalp rash, edema of the eyelids, forehead, or neck, eczema of the scalp or neighboring skin, facial swelling, or even a more generalized rash		(20)
Hair perms	Cocamidopropyl betaine (CAPB)	Allergic contact dermatitis, irritant reactions, occupational contact allergy: hand eczema	Surface cleaner	(17)

### The structural and physical characteristics of the hair shaft and scalp

**Figure 1:**
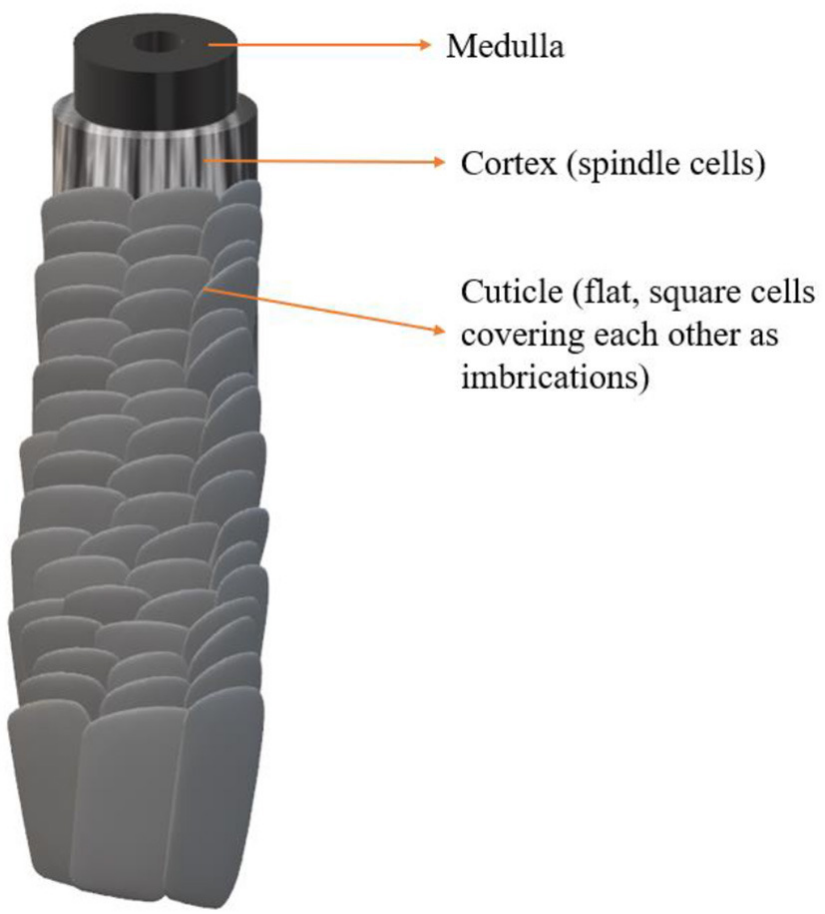
The ultrastructure of the hair shaft contains the medulla, cortex, and cuticle from the inner layer to the outer layer.

Hair integrally consists of the hair root and shaft (3). The hair shaft projecting beyond the scalp surface looks naturally glossy, smooth, pigmented, and flexible, as well as can withstand shearing forces (21). Structurally, the hair shaft is roughly divided into three layers: cuticle, cortex, and occasionally the medulla, from superficial to central. The central medulla is located at the core of the dark hair shaft, usually as a hollow core, and it will disappear as the hair shaft turns gray. The structure of keratins in the medulla is β-sheet, while it is in the presence of α-helix in the other two layers. The cortex surrounded by a multilayered complex cuticle consists of fusiform cells, whose long axis is parallel with that of hair. The cortex cells are made up of and surrounded with microfibrils, which are comprised of fibrils (2–3 strings of α-keratins). Microfibrils are 7–8 nm in diameter and are grouped into macrofibrils. All these fibers are either embedded in the cellular matrix or wrap the cortex cells from the surface. Cells are fused tightly with one another by the disulfide bond between keratins, determining the form of hair (22). Moreover, the varying colors of hair are dependent on natural color pigments in the cortex (3). Cells of the cuticle layer are flat and square, extensively overlapping with each other to present as imbrications. Healthy hair has six–eight layers of cuticle cells and is closely adherent to the inner cortical layer of the hair shaft, and each cell is attached to the nearby cells, only to have 1/6 of its surface as the free edge to point at the tip of hair (22, 23). Those tightly packed cells made up of proteins, lipids, and polysaccharides in the cuticle are colorless. The structure characteristics of the cuticle provide protection from external environmental factors (acts as an impenetrable barrier to external environmental damage), and when intact, the cuticle keeps the hair surface smooth and glossy (21). In terms of hair cosmetics such as hair dyes and perms, the cuticle plays an important role in regulating the entry and exit of chemicals/water to and from the cortex. On the other hand, the cuticle is impaired in procedures that require disruption of the cortex, such as bleaching, permanent coloring, and perming. Another desirable structure is the cell membrane complex (CMC). CMC is a hydrophobic non-cellular layer, seen in the gap between cuticle cells and cortex cells. This lipid layer contributes to the fusion of the cuticle and the cortex, the shelter from friction and dryness. The most important lipid constituent of it is 18-methyleicosanoic acid (18-MEA), which can only be seen in the cuticle layer (22) (Figure 1).

Mechanically, the depth of penetration of the hair shaft through temporary, semi-permanent, and permanent hair dyes, as well as perming agents, varies owing to the structural characteristics of the hair shaft. In the case of permanent coloring agents, the smaller molecules of dye precursors easily penetrate the cortex and undergo oxidation to form large colored molecules trapped inside the hair cortex with a lower possibility of diffusing out in subsequent washes. In contrast, the slightly larger molecules of semi-permanent hair dyes are capable of penetrating the cortex, but they are easily removable by shampooing. Worse still, temporary hair dyes are unable to penetrate the cortex because of larger molecules of substances. In other words, temporary and semi-permanent products impart color by relying on van der Waals forces for adhesion rather than chemical reactions activated in the cortex (3).

**Figure 2:**
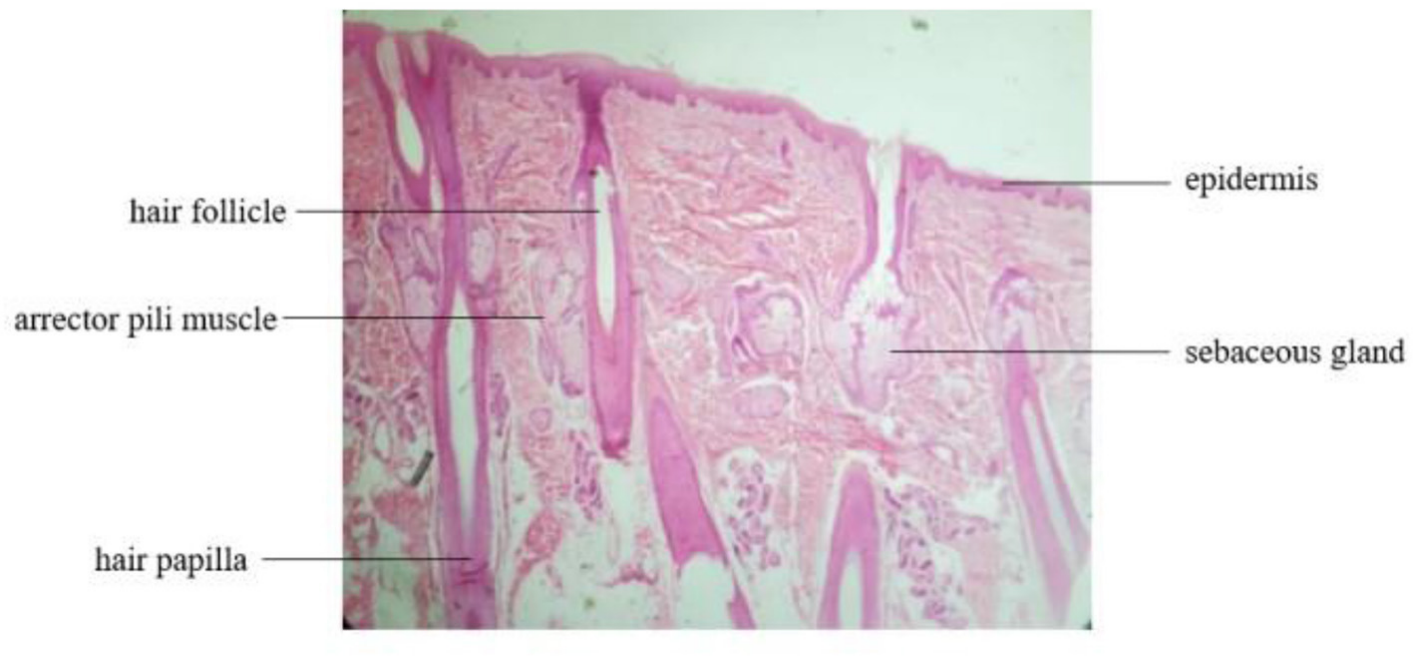
Histological structure of the scalp depicted contains the epidermis and dermis.

Similarly, the scalp is also a part of the skin and has the general structure of the skin, but it has some unique structural features compared to what is commonly thought of as skin (Figure 2). First of all, the hair follicles and glands of the head skin are much more than the skin of other parts, and the metabolic cycle is relatively shorter, so the secretion speed of its lipids will be faster. Meanwhile, due to the presence of hair covering and its anatomical location, the scalp is usually poorly cleared out and examined (24). A moist, lipid-rich environment is a breeding ground for microbes, which can lead to scalp problems such as dandruff (25). Although the skin on the head has more affiliate structures, it is actually thinner than the skin on the face (26). It is just thicker than the skin around the eyes on the whole body. Therefore, the scalp is very sensitive, which makes it easy for itching and other skin problems to occur when stimulated (27). The scalp ages fast, and an aging scalp will further affect the growth of hair follicles and hair. In addition, the scalp is one of the richest blood supplies throughout the body. A compression or blockage of the main scalp arteries is regarded as a likely cause of migraine pain (28). The tissue under the scalp is rich in blood vessels and nerves—the hair follicle is highly innervated with four types of specific nerve endings (27)—so the condition of the scalp is susceptible to people's emotions, stress, and so on.

### Impairment in the hair shaft

**Figure 3:**
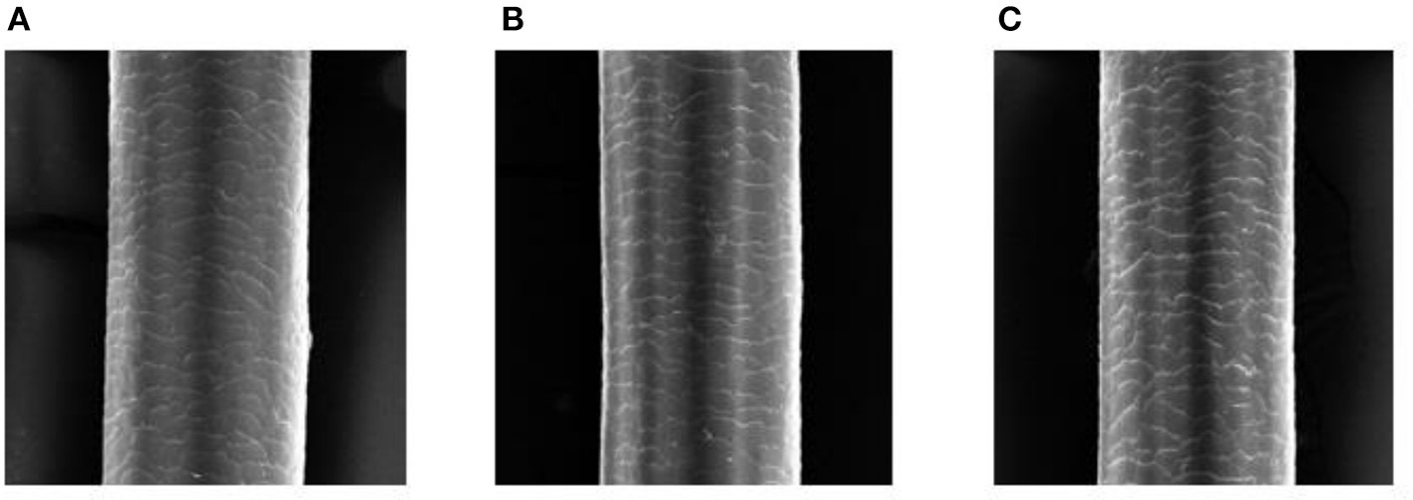
Electron microscope (EM) pictures of the natural, dyed, and permed hair shaft are depicted here. Picture **(A)** shows the morphology of the natural hair shaft under EM: Cuticles of the hair shaft are flat and covered with each other like imbrications. In comparison with picture **(A)**, picture **(B)** shows the morphology of the dyed hair shaft under EM: The hair cuticle cells are slightly disordered; layers of cuticles are damaged and broken, and the cortex is exposed. In comparison with picture **(A)**, picture **(C)** shows the morphology of the permed hair shaft under EM: The hair cuticle cells are disordered; the edge of hair cuticle cells is more evident and the hair cuticle cells are more damaged. Pictures **(B, C)** show the mild difference between dyed hair and waved hair: Waved hair has more damaged and disordered hair cuticle cells than dyed hair.

Hair cosmetic products are divided into two categories as follows: category 1 are those that function on the exocuticle, which can be washed off easily; and category 2 are those that work on the cortex or that alter the structure of the hair, making a long-lasting difference to natural hair (21). Extensive researches reveal that the latter has a significantly negative effect on the hair shaft. Permanent and semi-permanent hair dyes and perms are representatives of the category 2 hair cosmetic products. The harm they do to the hair shaft occurs not only at the molecular level but also at the cellular level: cell membrane complex (CMC), melanin granule and cuticle, and cortex, all of which can be damaged to varying degrees after being treated with hair perms or dyes. On the other hand, the impairment resulting from permanent hair perms and/or dyes involves structural damage, chemical constituent disorder, and impaired physical properties. The characteristic physical and chemical damage is shown in Figure 3 via electron microscope (EM).

Hair perms have an effect on breaking the disulfide bond in hair, which is the necessary step to reshape the hairstyle, but it is the main mechanism that is harmful to hair. In terms of physical and mechanical property changes of human hair resulting from all phases of hair perming, a certain amount of research has been performed (29, 30). Baus et al. revealed that strongly enhanced penetrability after perming was reflected by the mean penetration depth for fluorescein of 25 μm compared to 5 μm for natural fibers. In terms of chemical structure, perming of hair impaired binding characteristics of keratins (Ker) with negligible effects for preactivation, whereas unmodified and preactivated keratin-associated proteins (KAP) showed results comparable to natural hair (31). Straightening is also called lanthionization. In this procedure, at least 35% of cysteine was transformed to lanthionine, resulting in hair changing from frizzy to straight. The effect is attractive, but its side effects need to be paid attention to. After straightening, the hairs can lose protein and moisture, as well as tensile strength (21). The microcosmic reasons for this phenomenon, as said, are the damage to the cuticle, cortex, and melanin granule. Under EM, normal hairs have cuticles consisting of more than six layers of cells, covering one another, while the cuticles of hairs treated by perm curl up or break off. Melanin granule damage can be evaluated through a EM (Figure 3). Hairs treated with perms have fewer melanin granules than the control group (32). The majority of the damage mentioned above is due to mercaptoacetic acid/ammonium thioglycolate, the main element of a perm. There are two mechanisms for hair coloration: direct dyeing and mordant dyeing. In brief, the hair dyeing process can be divided into two steps: (i) diffusion of dye molecules from the dye bath to the keratinous hair fiber; and (ii) formation of chemical bonds (hydrogen, ionic, and covalent bonds) between the carboxyl or hydroxyl groups present in the dye molecules and amino/sulfhydryl groups in hair keratin, with or without the aid of auxiliary mordanting agents (9).

During the process of perming hair, thermal treatment can also contribute to the loss of moisture and tryptophan degradation (33). Copper plays a role in hair damage: a trace of copper is added to perm as a catalyst, but copper functions as a catalyst in UV-induced/non-UV-induced damage mechanism too (34). Hair dyes damage hair shafts differently than perms, and the damaging effects of permanent hair dyes are widely known. The major harmful element of permanent hair dyes is the alkaline base that is used to increase the permeability of cuticles, which is oppositely destructive to hair structure. The principle of permanent hair dyes during the process of hair dyeing can be roughly categorized into three steps: swelling, penetration, and oxidation (3). Swelling of the hair cuticle induced by the combination of oxidizing and alkaline agents in the permanent hair dyes makes it easy for the subsequent penetration of the colorless precursors into the hair cortex. The colorless precursors undergo oxidation, which can bleach the natural melanin pigment in the hair cortex and then change into large colored molecules to remain within the cortex with the least possibility of diffusing out. However, the oxidation process of the colorless precursors does irreversible oxidative damage to the hair shaft. Specifically, the hydrogen peroxide in the oxidation process accentuates the injury to the surface and diminishes the strength of the hair shaft (35). Relevant studies have demonstrated that the hair shaft can sustain oxidative damage with permanent hair dye use, which can be characterized by hair breakage. The impairment of the hair shaft can be assessed through multiple parameters: alterations in the surface architecture of the highly keratinized fibers, physical properties such as mechanical strength and moisture content, or morphological changes and variations in different structural components of hair fibers (31).

The transmission electron microscope is also used to assess the dyes' damage to hair. The negative effect of dyes is similar to perms: layers of cuticles are damaged and broken, exposing the cortex. Through proteomic analysis, the proteins falling from virgin hairs indicate that chemical damage occurs in the cortex layer and even deep in the fiber, changing protein conformation and cleaving disulfide bonds and keratin (36). Furthermore, the opening of the cuticles leads to the oxidation of the melanin granule, weakening the protection against optical radiation (37). In addition, there is a racial difference between perms and dyes: the perms are more harmful to Asian and African hair cuticles than white Europeans, and the dyes are more harmful to Asian and white European groups.

With EM, damage to CMC is detected. CMC has good tolerance to dye, but after several times of the coloring treatment, Asian hairs' CMC proves to be more probable to get bulging compared to white European and African's hair. In addition, dyes can remove the 18-MEA layer, an ingredient of CMC, making hair coarse and dull (21, 32). Cationic dyes are a sort of semi-permanent hair dyes, some of which possibly contain copper, thus having the effect to damage hair by participating in UV-induced/non-UV-induced mechanisms.

**Figure 4:**
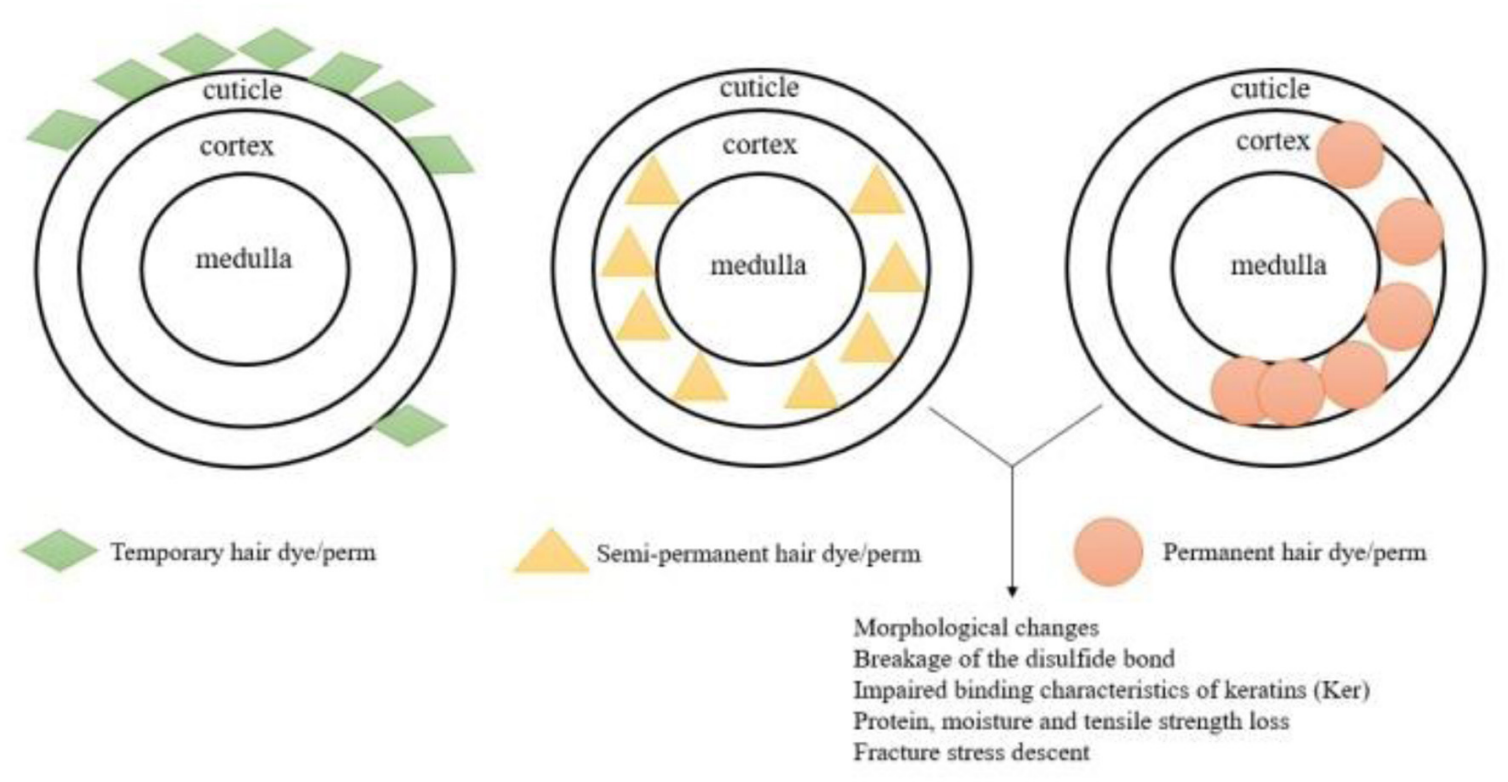
Mechanisms of three categories of hair dyes/perms are depicted. Molecules of temporary hair dyes/perms are adhered to the cuticle of the hair shaft and can be washed off through several shampoos. Ingredients of semi-permanent hair dyes/perms penetrate the cuticle and cortex, but owing to the small volumes, the duration of such hair chemicals cannot last for a long time. Small molecular precursors are restrained in the cortex and then transformed into larger ones so that they cannot be washed off easily. Structural damage of the hair shaft can be observed by some macro or micro indicators: morphological changes, breakage of the disulfide bond, impaired binding characteristics of keratins (Ker), protein, moisture, tensile strength loss, and fracture stress descent.

Generally, procedures that require the disruption of the cortex through chemical hair product (hair dyes and hair perms) usage damage the structural integrity of the hair. By using some techniques, the reduction in the tensile strength of semi-permanent or permanent colored or waved hair can be elucidated. Structural components of hair are essential to physical characteristics. By using Raman spectroscopy, the structure of cross-sections at various depths of colored or waved human hair, and through EM, the disturbance of the macrofibril (the microfibril and matrix) existing in the cortex region are shown to be associated with the reduction in tensile strength of semi-permanent or permanent colored or waved hair (21, 29). Despite the reduction of tensile strength, other physical characteristics such as alkali solubility, fracture stress, and water absorption expansion are changed compared to the natural hair (Figure 4).

### Impairment in the scalp

More than 2000 years ago, women in ancient Egypt learned to perm. Since 1872, with the advent of a special perm and with the development of the times, hair coloring and perms have become fashionable. Behind people's pursuit of fashion, there is sometimes a hidden danger to health. In recent years, it is not uncommon to see scalp injuries caused by hair coloring or perming. No matter whether the hair is colored or permed, the caustic damage can be divided into chemical damage and physical one (38), which will be differently explained as follows compared to the impairment in the scalp.

#### Physical damage

In the process of hair dyeing or perming, physical damage mainly refers to thermal damage (38, 39). At present, cold ironing technology on the market has developed relatively mature; many people will choose cold ironing when perming, therefore, thermal damage is more common in the hair dyeing process. Thermal injuries are mainly caused by the direct contact between the hot aluminum foil or hot hairdryer and the scalp during the hair coloring process (38), resulting in scalp burns. At the same time, one thing that cannot be ignored is that if the proportion of each component in the hair dye or perm agent is not appropriate, when they react on the scalp (40), they also give off quite a bit of heat. Due to the sensitivity and fragility of the scalp, the heat will make the person feel an intense burning sensation (40) and may burn the scalp. It is also another source of thermal damage.

According to the extent of burning, thermal injury symptoms are also different. Mild symptoms include erythema and blisters (40), and more serious symptoms can be severe burning scalp inflammation (41). If the thermal injury is serious, it will inevitably cause damage to the accessory structure of the skin. The hair follicles at the damaged site cannot be regenerated after being destroyed (38). The phenomenon of local hair loss, namely, cicatricial alopecia, occurs and the hair at the scar cannot grow back, which is one of the most serious complications of scalp burn. Cicatricial alopecia not only causes chronic pain and discomfort but more importantly, it affects the appearance of the patient, which in turn affects the patient's psychology. Some people may choose to dye or perm their hair to pursue beauty, but once such a condition occurs, it will cause a more severe psychological blow to these people. Analysis of microscopic images of the scalp functionalized with fluorescent dye chemicals demonstrated that nanoparticles can be bound to the keratin of the hair shaft and localized on the skin surface and even penetrate to the adjacent hair follicle (42).

#### Chemical damage

In the process of dyeing or perming, dye and perm agents contain oxide and alkaline composition to some extent. In addition, some individuals may be allergic to a particular component (41), and some people can suffer from toxic reactions affecting the health of the scalp due to its special structure which is easily penetrated by chemicals. High concentrations of oxides contacting the scalp directly can cause coagulation necrosis of the scalp tissue while denaturing proteins and causing cell damage through cytotoxicity (40).

In the long-term stimulation of these substances, the role of the scalp as the skin acting as the first human immune defense barrier will be weakened. Furthermore, owing to this long-term bad stimulation, hair scalp is likely to begin dysplasia and even canceration (41). Clinical cases reported combined immediate and delayed hypersensitivity induced by different types of PPD and PTD-containing hair dyes, which may arouse caution for patients to avoid recurrent systemic anaphylactoid reactions for oxidative hair dyes (43).

### The potential of inducing cancer

The hair dyes and perming agents that are predominately used contain chemicals recognized as carcinogens potentially relevant to several types of cancers, such as breast cancer, prostate cancer, hematopoietic cancer, ovarian cancer (44), and bladder cancer. In epidemiological studies, cancer incidence/mortality associated with personal use of hair products has been shown by lines of evidence for relative risk (RR) estimates along with corresponding 95% confidence intervals (CIs). In contrast, some appreciable excess risk of cancer among personal hair product users has been excluded by some statistical analysis.

**Figure 5:**
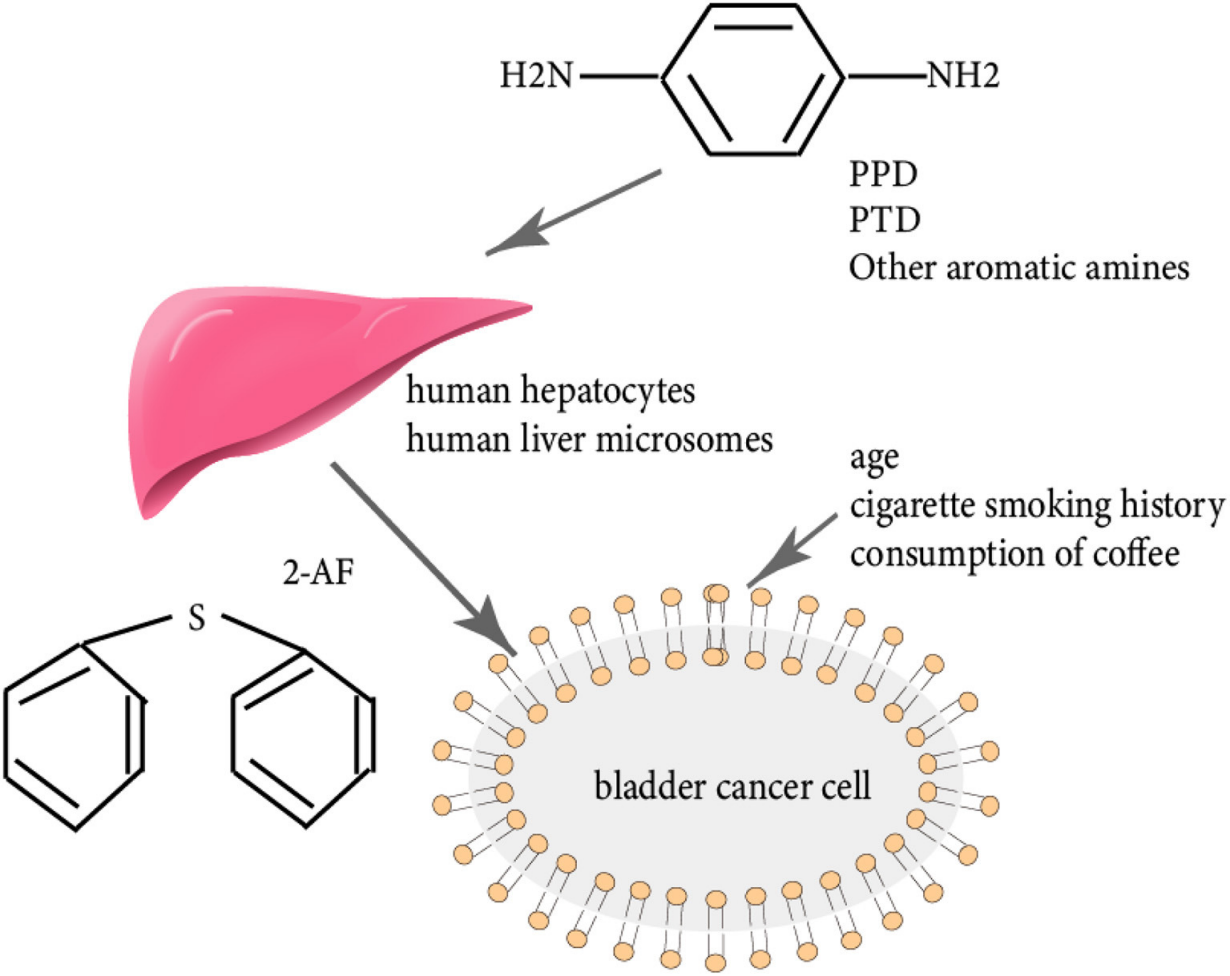
The picture depicts the possible carcinogenesis mechanism of chemicals in hair products. Aromatic amines in hair products such as PPD and PTD are first converted into bladder cancer carcinogens known as 2-aminofluorene (2-AF). 2-AF associated with other risk factors including age, cigarette smoking history, and consumption of coffee potentially induce bladder cancer.

Declarations of linkage between personal hair dyes/perming agents and the risk of cancers are controversial in general. Heavy metals such as Pb, Cd, Co, Cr, and Ni were included in 36 samples of hair dyes from four best-selling brands in the Kashan market (Iran). Carcinogenic health risk assessment results showed that a low level of exposure to heavy metals in hair dye users can induce potential cancers among users of Iranian ethnicity (45). A large prospective cohort study, conducted by the epidemiology branch of the National Institute of Environmental Health Sciences (NIEHS), found a positive association between chemical hair products and breast cancer risk, especially in black women. Over a total of 386,338 person-years, 2,794 incident breast cancer cases were reported by using multivariable Cox regression models adjusting for likely confounding variables, which supported the associations of hair chemicals with breast cancer risk [prospective cohort hazard ratio (HR), 1.09; 95% CI, 1.01–1.17] and a stronger association (HR, 1.45; 95% CI, 1.10–1.90) (46). A study in carcinogenesis suggested the risk of breast cancer with respect to subjects' use of chemical hair care products, which had some differences by race (47, 48), various shades of hair coloring, and exposure level. Specifically, an increased risk of breast cancer emerged among black women despite lower exposure, which is even more evident in participants using darker shades of hair dye than controls. The conclusion was supported by the data of a case–control study including 2,280 breast cancer case patients (1,508 black and 772 white) and 2,005 control subjects (1,290 black and 715 white). Similar to the linkage between increased risks of non-Hodgkin lymphoma and leukemia and the use of hair products, studies of breast cancer risk have yielded conflicting results (49). The higher risk of breast cancer in black women was also evaluated by a national prospective cohort study enrolling 46,709 Sister Study participants with the result that permanent dye use was associated with 45% higher breast cancer risk in black women (HR = 1.45, 95% CI: 1.10–1.90) and 7% higher risk in white women (HR = 1.07, 95% CI: 0.99–1.16; heterogeneity *p* = 0.04) (47). However, considering the valuable outcome of the study, several points such as the more accurate interval between use and breast cancer (BC), other various variables, and confounding factors need to be addressed when conducting such large-scale studies (47). Some research concluded that hormonally active and carcinogenic compounds could be contained in hair products (known as aromatic amines) (47). Breast tissue is extremely susceptible to these chemicals in adolescence. A large prospective cohort study including Sister Study participants (ages 35–74 years) who had completed enrollment questionnaires (2003–2009) on the use of hair products drew the plausible conclusion that frequent use of dyes and perms during adolescence was associated with a higher risk of premenopausal (HR = 2.11, 95% CI 1.26–3.55 and HR = 1.55, 95%CI: 0.96–2.53, respectively) but not postmenopausal breast cancer (HR = 0.99, 95% CI: 0.76–1.30 and HR = 1.09, 95% CI: 0.89–1.35, respectively) by using Cox proportional hazards regression (50). Specifically, breast tumor clinicopathology features associated with hair product exposure have increased odds of poor differentiation, tumors >2.0 cm vs. <1.0 cm (OR = 1.82, 95% CI: 1.23–2.69), higher tumor grade, and lymph node status in the overall sample. Building on such prior studies, breast cancer prognostic indication is predicted and guidance of risk reduction strategies is possible to be denoted (51). Nevertheless, the more precise possibility of different clinical types of breast cancer in users of permanent hair dyes varies warrants further investigation as increased breast cancer risk and mortality has been estimated for estrogen receptor-negative breast cancer, progesterone receptor-negative breast cancer, and hormone receptor-negative breast cancer (5). Additionally, other studies reported that an increased risk of bladder cancer was due to the use of hair dyes (3), straighteners/relaxers, and perms, although such evidence was not found in some other studies (5). According to a meta-analysis of 17 studies (15 case–control and two cohort studies) and over 8,500 cases, the pooled RR of bladder cancer for the personal use of any type of hair dyes was 0.93 (95% CI, 0.82–1.05) in the presence of moderate heterogeneity among the studies (*I*^2^ = 34.1%, *p* = 0.07) and more specifically, similar RRs were found for female subjects (RR = 0.95) and male subjects (RR = 0.81) compared with no use, as well as the random-effect pooled RR for the personal use of permanent hair dyes was 0.92 (95% CI, 0.77–1.09). The association between bladder cancer and hair products was observed with consideration of several potentially eligible exposure aspects such as type of dyes, color, duration, and frequency of lifetime applications, which may bias the consistency of the results (52). Conversely, inconsistent results have been produced by investigations in a review (containing 11 case–control studies and three cohort studies) (53). Assessment studies by Souza et al. investigated the mutagenicity property of the compounds generated by the oxidation reaction involving ptoluenediamine (PTD) and p-aminophenol (PAP), precursors detected in permanent hair dyes. The mutagenicity assay showed that hazardous sub-products presented mutagenic activity by using Salmonella typhimurium YG1041 strains, with and without metabolic activation (S9), and the result was positive when the bacterium Salmonella typhimurium YG1041 was in the presence of S9, with MEC2 of 10.0 μg μl^−1^ (51). The detected mutagenicity illustrates the cancer potential of commercial hair dyes (54). Additionally, some mechanistic studies on hair product carcinogenicity revealed that concerning the carcinogenesis of N-hydroxylamine in the development of bladder cancer, N-oxidation leading to the formation of an N-hydroxylamine in the human hepatocytes or human liver microsomes plays a significant role. PPD is the most important arylamine in many oxidative hair products, which is converted into a proximate bladder carcinogen known as 2-aminofluorene (2-AF) (55) (Figure 5). More epidemiological risk assessments of those who are diagnosed with bladder cancer and were exposed to aromatic amines concluded occupational and non-occupational risk factors such as age, cigarette smoking history, and the consumption of coffee (56).

## Interventions for allergy to hair dyes

### Novel alternative hair dyes to hazardous para-phenylenediamine

Para-phenylenediamine (PPD) along with a coupler (a kind of catalyst) and an oxidizing agent, and other related members of the aromatic amine family are the main compounds of most of the permanent hair dyes, characterized by their low molecular weight, the capability of penetrating the hair shaft and follicle, and the strong ability to bind protein (57). However, the results of murine local lymph node assay (LLNA) in response to paraphenylenediamine in both intralaboratory and interlaboratory tests are positive (58). Furthermore, statistics reveal that the rate of positive path tests designed for PPD is ~4% in Europe, 4.3% in Asia, and 6.2% in North America (14). PPD is an ideal and potent contact allergen clinically. McFadden et al. elucidated the prevalence of hair dye products during the 20th century and the frequency of allergic contact dermatitis (ACD) occurring in the population of a large age spectrum and stated that no satisfactory or widely accepted alternatives to the aromatic amine agents are available for use in permanent hair dye (57). Additionally, ACD induced by hair dye is a growing concern both for consumers and the cosmetics industry.

A synthetic formulation that excludes PPD may help individuals who are sensitive avoid traditional formulations that induce ACD. To this end, herbal or plant dye products such as henna were delineated as alternative hair dyes with low allergic potential (59). Therefore, recently, an increasing number of studies on alternative hair dyes to hazardous PPD have been done to foster advances in reducing the incidence rate of ACD induced by PPD. Earlier, Goebel et al. developed the PPD-derivative 2-methoxyethyl p-phenylenediamine (ME-PPD), which displayed reduced sensitization potential in the cysteine Direct Peptide Reactivity Assay (DPRA) compared to PPD and PTD in the absence of enzymatic oxidation (60). ME-PPD has excellent hair coloring performance and significantly reduced sensitizing properties compared to PPD and PTD, through the introduction of a methoxymethyl side chain (60), which may inhibit binding to the CD86 costimulatory marker on antigen-presenting cells that is necessary for T-cell activation (61). Moreover, dermal dendritic cells (DCs) activation was observed, LLNA showed a lower sensitization potential (13), and cross-elicitation of ME-PPD in PPD-allergic volunteers was lower (62). The results of pretests with 2-methoxymethyl-paraphenylenediamine-containing hair dye products among PPD/PTD-allergic individuals conveyed the message that the introduction of ME-PPD into commercial hair dye products possibly benefits consumers, hairdressers, and manufacturers. However, a (cross-)elicitation rate of ME-PPD containing hair dyes remains ~30%, which may limit the subsequent progression of ME-PPD (63). Venkatesan et al. (13) investigated novel hair dyes, PPD6 and PPD7, with electron-withdrawing properties at the ortho position of PPD and assessed their hair dyeing efficacy, percutaneous penetration, and toxicity profiles, and the results indicated they were excellent candidates. It is reported that PPD6 and PPD7 are synthesized through the strategic introduction of electron-withdrawing esters at the ortho position of the PPD monomer and PPD dimer, which may overcome the hazard potential of PPD. Furthermore, PPD6 is the intermediate product in the synthesis procedure of PPD7. The results of the hair dyeing study, low ΔE values under the chromometer assay, showed that the hair dyeing efficacy of PPD6 and PPD7 under oxidative conditions were preserved similarly to PPD. Additionally, the increase in molecular size of the PPD derivatives, PPD6 and PPD7, contributed to lower penetration in comparison to PPD, ME-PPD, and PTD in the skin permeation study. More importantly, cytotoxicity assays on HaCaT cells of PPD6 and PPD7 presented the highest IC50 (i.e., the lowest cytotoxicity) and no expression of inflammatory IL-8, which demonstrated the decreasing cytotoxicity caused by PPD6 and PPD7 toward skin cells (13) (Table 2).

**Table 2:** Some potential alternatives of hair chemicals, especially hair dyes, include an alternative variety of hair products and other synthesized compounds.

**Order**	**Interventions**	**Function**	**References**
1	Herbal or plant dye products	Replace hair chemicals	(59)
2	2-methoxyethyl p-phenylenediamine (ME-PPD)	Alternative to PPD	(60–63)
3	PPD6 and PPD7	Novel alternative to PPD	(13)
4	A barrier of petrolatum	A barrier preventing hair dye dermatitis	(64)
5	A vitamin C-containing cream	Antioxidative agents preventing hair dye dermatitis	(65)
6	Cationic surfactant (stearalkonium chloride, cationic polymer)	Protect the hair shaft	(66)
7	Conditioning agents (protein)	Protect the hair shaft	(66)

Additionally, other preventive measures are listed in the table.

### Preventive interventions

During hair dyeing preparations, to prevent possible ACD, it is recommended that the hairdresser should apply a barrier of petrolatum to minimize the lateral spread of the dye (64). Applying 0.05% clobetasol propionate foam to the affected areas twice a day for 3 days before coloring can evidently prevent hair dye dermatitis (67). Notably, permanent hair dyeing has been reported to be based on the oxidative properties of aromatic amines such as PPD, which may illustrate its allergenic potency. Therefore, it is possible to reduce contact sensitization to hair dyes by eliminating the allergenic signal deriving from the oxidation of hair dye precursors not otherwise consumed during the hair-coloring process. To this end, Basketter et al. pretreated glabrous skin with antioxidative agents such as a vitamin C-containing cream, and the results suggested that the ACD level was significantly reduced on approximately three-quarters of the test sites (65) (Table 2).

## Interventions for hair shaft damage caused by hair dyes

Hair damage caused by hair dye can be assessed by protein loss. The validated bicinchoninic acid (BCA) method was used to evaluate the hair protein loss caused by permanent hair dyes, and the results showed that hair dyes based on ammonium thioglycolate and guanidine hydroxide did not increase protein loss in dyed hair (68). Ahn et al. examined the ultrastructural changes of hair shafts through sequential scanning and transmission electron microscopy at the precoloring and postcoloring periods and concluded that the application of an acidic pH solution (an acidic hair care product decreasing the permeability of the hair cuticle) in the rinse immediately after use of the hair dye helped accelerate mild hair damage recovery. Additionally, accelerated restoration of the hair is possible if hair care products containing cationic surfactants such as stearalkonium chloride and cationic polymer, and conditioning agents such as protein are applied during the period of hair dyeing (66).

## Summary, perspectives, and future directions

Hair products including hair dyes, perms, and straighteners/relaxers are common cosmetics used among people of different ages throughout the world. Commercialized hair highlighting products can be categorized by the chemical ingredients in the formulation. The whole chemical process of highlighting the hair is a chemical reaction that calls for three classes of reactants: primary intermediates, couplers, and an oxidant. PPD is the most significant component among the three classes of reactants (66). Any chemical process of cosmetic treatment for hair dyeing or perming affects the chemical structure of the hair shaft, and consequently induces structural damage, chemical constituent disorder, and impaired physical properties of the hair shaft. Structurally, the human hair shaft consists of the main cortex along with a central axial medulla and an external cuticular layer (21–23). Hair damage is presented by microscopical hair analysis through scanning or transmission electron microscopy on the microstructure level, correlating with hair morphology variation (66). The hair-highlighting mixture pooled onto the scalp may cause allergic contact dermatitis, irritant contact dermatitis, scalp burns, or more serious disease. Some light-to-moderate ACD cases are neglected. This review has given an overview of the potential cancer risk related to the application of personal hair dyes or perms. Several studies have estimated the association between hair dye and perm use and breast and bladder cancer risk, as well as potential differences in associations by ethnicity, although some existing studies show null carcinogenic effects in humans. While the current study results are similar to other published data, an exploration into the improvement of formulations in hair products is hoped to relieve these potentially serious side effects and advance our understanding in this area.

## Author contributions

YH: conceptualization, writing—original draft preparation, and picture (using software). YC: writing—original draft preparation, methodology, and table. BN: writing—reviewing and data curation. All authors contributed to the article and approved the submitted version.
